# Decreased insulin dose-adjusted hemoglobin A1c in adults with cystic fibrosis-related diabetes treated with elexacaftor-tezacaftor-ivacaftor

**DOI:** 10.1016/j.jcte.2025.100407

**Published:** 2025-07-02

**Authors:** Espérie Burnet, Deborah Grunewald, Etienne Larger, Florence Camus-Bablon, Catherine Eisenhauer, François Mifsud, Clémence Martin, Isabelle Honoré, Reem Kanaan, Nicolas Carlier, Johanna Fesenbeckh, Helen Mosnier-Pudar, Pierre-Régis Burgel

**Affiliations:** a Respiratory Medicine and National Reference Cystic Fibrosis Reference Center, Cochin Hospital, Assistance Publique Hôpitaux de Paris (AP-HP), Paris, France; b ERN-Lung CF Network, Frankfurt, Germany; c Diabetes Department, Cochin Hospital, Assistance Publique Hôpitaux de Paris (AP-HP), Paris, France; d Université Paris Cité, Institut Cochin, Inserm U1016, Paris, France; e Department of Epidemiology and Training, Epicentre, Paris, France

**Keywords:** Cystic fibrosis related diabetes, Cystic fibrosis, Diabetes, Elexacaftor-tezacaftor-ivacaftor, Insulin dose adjusted hemoglobin A1c, Glycemic control, Insulin

## Abstract

**Background:**

Elexacaftor-tezacaftor-ivacaftor (ETI) became available for adults with cystic fibrosis (CF) in 2019, but its impact on CF-related diabetes (CFRD) remains unclear.

**Methods:**

A single-center retrospective cohort study was conducted among adults with CFRD to examine the change in insulin dose-adjusted Hemoglobin A1c (IDAA1c). Linear mixed effects model (LMEM) analysis was used to investigate the change in IDAA1c between baseline and 24 months of follow up, comparing an ETI-treated group to an unexposed group. Baseline values were those documented at treatment initiation for the ETI-treated group and in March 2020 (±3 months) for the unexposed group.

**Results:**

A total of 49 adults were included, 39 were treated with ETI and 10 were not. Median [Interquartile range] time since CFRD diagnosis at baseline was 13 [7–18] and 14 [10–18] years, respectively (p = 0.610). In the ETI-treated group, mean weight increased by a 4.44 kg (95 % Confidence Interval, 95 %CI: 3.08 to 5.79, p < 0.001), insulin total daily dose decreased by 5 units (95 %CI: −9 to 0, p = 0.033), and hemoglobin A1c (%) decreased by 0.65 points (95 %CI: −0.96 to −0.34, p < 0.001). No change was observed in the unexposed group. LMEM analysis found a numerically significant association between ETI and decreased IDAA1c, estimated at −1.14 points (95 %CI: −2.35 to 0.06, p = 0.067) after adjusting for age, sex, time since CFRD diagnosis and the introduction of Metformin.

**Conclusion:**

A numerically significant association between ETI and IDAA1c decrease was observed in adults with established CFRD after 24 months of treatment, suggesting ETI contributed to improved glycemic control.

## Highlights

• Cystic Fibrosis (CF) Related Diabetes (CFRD) affects 30% of people with CF (pwCF)
• Elexacaftor-tezacaftor-ivacaftor (ETI) has been available for most pwCF since 2020.
• A retrospective cohort study compared 39 ETI-treated pwCF to 10 unexposed pwCF.
• The outcome variable was the change in Insulin Dose Adjusted Hemoglobin A1c (IDAA1C)
• ETI was correlated with a 1.14 (p = 0.067) point decrease in IDAA1C after 24 months.

## Introduction

Cystic Fibrosis-Related Diabetes (CFRD) is the most common non-pulmonary complication in people with cystic fibrosis (pwCF), with an estimated prevalence in 2022 of 20 % to 50 % among adults [[1], [2], [3], [4]]. It is known to contribute to lung function decline and has been associated with increased morbidity and mortality [[5], [6], [7]]. The pathophysiological mechanism is two pronged, combining both insulin production deficiency, due to pancreatic cytotoxicity and fibrosis, and insulin resistance [8], to which frequent respiratory infectious exacerbations may contribute [9]. Clinical guidelines have historically recommended annual screening among children starting at age 10 and the early introduction of insulin [10].

The availability of elexacaftor-tezacaftor-ivacaftor (ETI), a triple combination of cystic fibrosis transmembrane conductance regulator (CFTR) modulators [11,12] has introduced a paradigm shift in the care and expectations of pwCF [13]. ETI has been shown to increase respiratory function and quality of life, while reducing the frequency of respiratory exacerbations [[11], [12], [13]]. Although ETI is also known to be associated with an increase in weight, its impact on CFRD remains poorly understood, in part due to the interaction between insulin dose, weight and Hemoglobin A1c. Glycemic control was not investigated as an endpoint in large Randomized Control Trials (RCTs) or in real-world studies evaluating the effectiveness of ETI [11,12,[14], [15], [16], [17]] and a limited number of observational studies with a small number of participants have evaluated the impact of ETI on CFRD over time, with conflicting results [[18], [19], [20], [21]].

To our knowledge, no study has evaluated the association between ETI and glycemic control by comparing trends over time among adults with established CFRD between ETI-exposed patients and an unexposed group. Given the known associations between ETI and increased weight on the one hand, which would lead to increased insulin resistance and needs, and between ETI and decreased systemic inflammation, which would lower them, evaluating glycemic control has proven difficult. Insulin dose-adjusted Hemoglobin A1c (IDAA1c), a compound measure accounting for variability in Hemoglobin A1c (HbA1c), insulin requirements and weight, has been used in the management of type 1 diabetes in non CF adults and children to estimate β-cell function, particularly in the honeymoon phase [22,23], and could also be used as a marker of insulin sensitivity. Hemoglobin A1c has not been recommended as a marker of CFRD progression because it does not capture glycemic variability around meal times, particularly post-prandial hyperglycemia, a distinct feature of CFRD [10]. C-peptide or mixed meal tolerance tests may be useful for assessing pancreatic reserve and endogenous insulin production but they are not conducted as part of routine care. By taking into account both insulin daily dose and weight, in addition to HbA1c, IDAA1c may therefore become relevant in monitoring CFRD.

The primary objective of the present study was to estimate the potential association between ETI treatment and improved glycemic control over 2 years of follow-up, using IDAA1c as an outcome measure, by comparing adults with CFRD who initiated ETI to an unexposed group, also with established CFRD. A secondary objective was to examine trends in IDAA1c, HbA1c, insulin daily dose and weight within in each group.

## Methods

### Study design and data collection

A single-center retrospective cohort study was conducted at Cochin Hospital adult CF center (Paris, France). Eligibility criteria included age ≥18 years, having a confirmed CFRD diagnosis, daily insulin treatment (long acting and/or short acting), and being followed in our center for a full 24 months after baseline date. CFRD was defined as having had a positive 2 h oral glucose tolerance test (OGTT) and being treated with insulin [24]. Patients on oral antidiabetic medications without insulin, organ transplant recipients and patients with gestational, cortico-induced or type 1 diabetes were excluded. Those who were transferred to another CF center, who received lung transplantation, or who died during the study period were also excluded.

Patients were grouped into an ETI-treated group, comprised of those who were treated with ETI for a full 24 months, and a non-ETI treated group, which included those that were not eligible to- or who interrupted ETI after 2 months or less due to adverse effects. Eligibility criteria to ETI during the study period followed those defined by the European Medicines Agency and allowed CF patients with at least one p.Phe508del CFTR mutation, approximately 83 % of French CF patients, to access treatment. At the time of the study, off-label use was also possible for patients with advanced lung disease through a Compassionate program, which allowed patients without eligible mutations to access ETI if they met specific clinical severity criteria [25]. Subjects categorized as ETI-treated initiated treatment between April 2019 and April 2022 and were followed for 24 (±3) months thereafter. Baseline values were those documented immediately prior to ETI initiation. In the unexposed group, baseline date was defined as February 2020 (±3 months) in order to cover the same observation period.

The main outcome variable was the mean absolute difference in IDAA1c between the 12 (M12) and 24-month (M24) time points and baseline (M0). Secondary outcome variables included the mean absolute change in HbA1c, total daily insulin dose, weight, and total daily insulin dose by weight. Total insulin daily dose was computed as the sum of long and short acting insulin units reported in patient medical files. HbA1c values were collected from hospital laboratory records over the previous 12 months, and the mean value was used in order to adjust for any measurements that may have occurred within 3 months of an exacerbation. IDAA1c was calculated as HbA1c (%) + [4 x insulin dose per kilogram per 24 h] [22].

Clinical and demographic data were collected retrospectively from existing electronic records at baseline, M12 and M24, and included: date of birth, sex, *CFTR* variants, year of CFRD diagnosis, weight, oral diabetes medications, existing CFTR modulator treatment at baseline, pancreatic insufficiency, presence of a continuous glucose monitoring (CGM) device, liver cirrhosis, as well as percent predicted forced expiratory volume in 1 sec (ppFEV_1_), number of IV antibiotic courses over the previous 12 months, pregnancy and enteral nutrition. When applicable, CGM data was retrieved for the previous 28 days. Data were collected at each time point ±3 months.

A power analysis using paired sample *t*-tests was conducted to determine the minimum sample size required to detect a statistically significant 1-point decrease in IDAA1c within each group after 24 months of follow-up, assuming a 5 % type 1 error and 80 % power, and yielded a total sample size of 50 in the exposed group and 40 in the unexposed group.

Eligible participants were informed of the study’s objectives and could decline to participate but written consent was not required as per French legislation. The study was approved by Cochin Hospital’s Ethical Review Committee for Publications (CLEP, approval number: AAA-2024-10023).

### Statistical analyses

Participant characteristics at baseline are presented as medians and interquartile ranges [IQR] and as numbers and percentages, as appropriate. Baseline characteristics were compared between groups using Mann Whitney U tests for continuous variables, assuming non-normal distributions, and χ^2^ test for discrete variables. A subgroup analysis among ETI treated subjects was conducted, using the same methods, to compare baseline characteristics between those who were treated with a previous CFTR modulator (ivacaftor in combination with tezacaftor or lumacaftor) and those who were modulator naïve at ETI initiation. Linear Mixed Effects Models (LMEM) were built to conduct univariate analyses of the change between time points (M12 vs baseline and M24 vs baseline) in each group for each variable under consideration. These models included “subject” as a random effect to account for individual variability and “time” as a fixed effect.

The association between ETI and reduced IDAA1c was also evaluated using LMEM analysis, comparing trends between ETI-exposed subjects and unexposed subjects. The model was built using a maximal to minimal backward stepwise approach. The initial model included ETI-treatment, time, sex, age, Metformin, time since CFRD diagnosis, CGM device, ≥1 Intravenous (IV) antibiotic courses per year and an interaction term for ETI:time to account for the absence of treatment at baseline. BMI was not included as a variable in the model as height was assumed to remain stable throughout the study period and in order to avoid collinearity with the outcome variable, which included weight. “Subject” was considered as a random effect and all other variables were considered as fixed effects. Continuous variables were centered on their means to ensure convergence and “time” was considered as a simple parameter in order to compare the change in outcome at M12 and M24 to baseline values. A second LMEM was built, using the same strategy, in the ETI exposed group only, in order to compare IDAA1c trends between subjects who were CFTR modulator naïve and those who were on CFTR modulator therapy prior to ETI initiation.

Variables were selected based on clinical criteria and tested for inclusion in the model with the goal of building the most parsimonious model. The Akaïke lnformation Criterion (AIC) and likelihood ratio tests (LRTs) were used to determine whether the removal of a variable from the baseline model improved model fit. With each iteration, AIC scores were compared to select the model with the lowest AIC, checking for the statistical significance of likelihood ratio tests, the normality of residuals distribution and model convergence. Parameter estimates were calculated using Restricted Maximum Likelihood estimations and statistical significance tests for model coefficients were performed using Satterthwaite approximations. All analyses were conducted using *Jamovi* software (version 2.4, GAMLj3 Suite for Linear Models. R package version 3.3.9) [26].

## Results

**Fig. 1:**
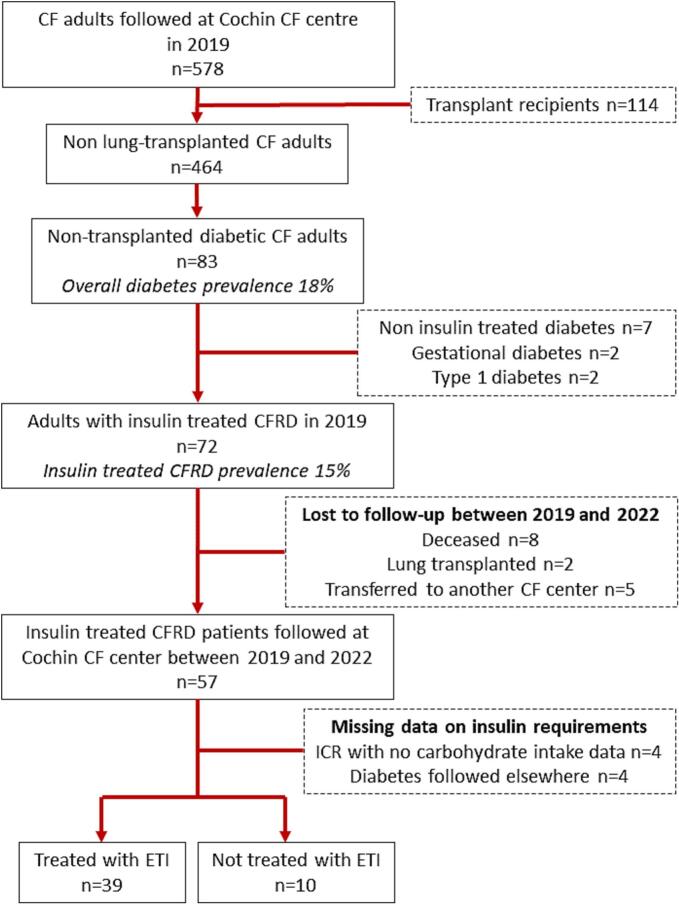
Inclusion flow-chart. CF: Cystic Fibrosis, CFRD: Cystic Fibrosis Related Diabetes, ICR: Insulin to Carbohydrate Ratio, ETI: Elexacaftor-Tezacaftor-Ivacaftor.

The prevalence of diabetes among the 464 non-transplanted adults with CF followed at Cochin CF center in 2019 was 18 % (n = 83); 72 were treated with insulin (15 %) and were eligible for inclusion. Between 2019 and 2022, eight died of respiratory failure and two received a lung transplant without having been treated with ETI. Another five were transferred to another CF center and four were followed elsewhere for CFRD. Two patients stopped insulin before ETI initiation. Another two were excluded due to missing data. None of the eligible subjects declined to participate. The study sample therefore included 49 adults with insulin-treated CFRD (see Fig. 1).

### Overall patient characteristics

Twenty-eight (57 %) of the 49 included subjects were p.Phe508del homozygous, 11 (22 %) were p.Phe508del heterozygous and 10 (21 %) had no p.Phe508del variant. Thirty-nine were categorized as ETI treated; 38 of which had a least one p.Phe508del variant. One N1303K homozygous subject obtained access to ETI through off-label authorization in October 2021. Seventeen had been taking a dual combination CFTR modulator at the time of ETI initiation; 14 were treated with lumacaftor-ivacaftor (LI), and 3 with tezacaftor-ivacaftor (TI).

The other 10 participants were categorized as unexposed to ETI. One p.Phe508del heterozygous participant developed severe myalgia within the first 2 weeks of ETI introduction and declined further treatment. One subject with the G551D/D1152H mutations was treated with Ivacaftor throughout the study period. The other eight were, and remained, CFTR-modulator naïve.

Baseline participant characteristics are presented in Table 1. Those who were not treated with ETI were somewhat older, with a median [IQR] age of 41 [33–55] compared to 35 [28–41] (p = 0.142) but time since CFRD diagnosis was similar in both groups, with a median [IQR] of 13 [7–18] months compared to 14 [10–18] (p = 0.610), respectively. At baseline, the difference between groups in terms of HbA1c, total daily insulin and weight was not statistically significant and median [IQR] IDAA1c was 8.5 [7.9–10.0] in the unexposed group and 9.3 [8.3–11.1] in the ETI-treated group (p = 0.747). None, in either group, were treated with oral antidiabetic agents in addition to insulin at baseline. All but one subject had 4 daily insulin injections throughout the study period; one rapid acting insulin injection with each meal and one long-acting insulin injection per day. Both groups were comparable in terms of baseline BMI, ppFEV1, number of IV antibiotic courses per year and sex distribution. All participants were pancreatic insufficient and only one patient required intermittent enteral nutrition.

**Table 1:** Characteristics of 49 included CFRD adults at baseline.

	CFRD adults not treated with ETI (n = 10)	CFRD adults treated with ETI (n = 39)	p-value	
Age (years)	41 [33–55]	35 [28–41]	0.142	
Time with CFRD (years)	13 [7–18]	14 [10–18]	0.610	
Female sex	6 (60 %)	19 (50 %)	0.524	
ppFEV_1_	54 [45–61]	53 [41–76)	0.719	
Weight (kg)	62 [48–67]	57 [51–64]	0.843	
BMI (kg/m^2^)	22.8 [21.0–24.1]	21.4 [19.9–22.5]	0.184	
Insulin dose (U/day)	22 [17–49]	36 [25–45]	0.292	
Insulin dose (U/kg/day)	0.43 [0.31–0.69]	0.58 [0.47–0.81]	0.292	
HbA1c (%)	7.2 [6.4–8.4]	6.8 [6.4–7.6]	0.441	
HbA1c (mmol/mol)	55.2 [46.2–69.4]	50.8 [46.5–59.6]	0.441	
IDAA1c	8.5 [7.9–10.9]	9.3 [8.3–11.1]	0.747	
Metformin use	0	0	1.000	
CGM device	0	10 (26 %)	<0.001	
IV antibiotic courses/year	2 [1.25–2]	1 [0–2]	0.149	
Non-ETI CFTR mod.	1 (10 %)†	17 (46 %)‡	0.010	
Pancreatic insufficiency	10 (100 %)	39 (100 %)	1.000	
Liver Cirrhosis	1 (10 %)	3 (10 %)	0.812	
Enteral nutrition	0	1 (3 %)	0.122	
*CFTR* variants	0	28 (72 %)	<0.001	
p.Phe508del/other	1 (10 %)*	10 (26 %)		
Other/other	9 (90 %)	1 (3 %)**		

Data presented as median [IQR] or n (%). Comparison between groups used the Mann Whitney *U* test for independent samples or χ^2^ test as appropriate. CF: Cystic Fibrosis; CFRD: Cystic Fibrosis Related Diabetes; ETI: Elexacaftor-Tezacaftor-Ivacaftor; ppFEV1: percent predicted Forced Expiratory Volume in one second; BMI: Basal Metabolic Index; U: units; HbA1c: hemoglobin A1c; IDAA1c: Insulin Dose-Adjusted hemoglobin A1c CGM: Continuous Glucose Monitoring; IV: intravenous; CFTR: Cystic Fibrosis Transmembrane Regulator; mod.: modulator

† one G551D/D1152H subject was treated with Ivacaftor throughout the study period. ‡14 were treated with lumacaftor-ivacaftor, and 3 with tezacaftor-ivacaftor.

* this patient interrupted treatment after 8 weeks due to invalidating myalgia. **N1303K/N1303K; this patient received ETI as part of the French Compassionate Program.

The subgroup comparison between CFTR modulator naïve subjects (n = 22) and those treated with TI (n = 14) or LI (n = 3) prior to ETI initiation showed that the median [IQR] daily insulin dose at baseline was higher in the CFTR modulator naïve group (39 U/day [32–54]) than in the LI/TI group (30 U/day [20–38], p = 0.023), though weight and HbA1c were similar. Baseline median IDAA1c in the CFTR modulator naïve group was therefore higher at 10.2 [9.0–12.1], versus 9.1 [7.7–9.4] in the TI/LI group (p = 0.011) (see Supplementary Table 1). The two groups were otherwise comparable in terms of age, CFRD duration and ppFEV1.

### Outcomes in the ETI treated group

In the 39 ETI treated participants, IDAA1c decreased by a mean of 0.87 points (95 % Confidence Interval (CI): −1.39 to −0.36, p < 0.001) at 12 months and by 1.44 points (95 % CI: −1.96 to −0.91, p < 0.001) at 24 months, representing a 9 % and 15 % decrease compared to baseline, respectively. Absolute HbA1c (%) decreased by 0.55 percentage points after 12 months of treatment (95 % CI: −0.87 to −0.26, p = 0.001) and by 0.65 percentage points at 24 months (95 % CI: −0.96 to −0.34, p < 0.001). The decrease in insulin total daily dose was not statistically significant at 12 months (p = 0.609) but was 5 units lower (95 % CI: −9 to 0) at 24 months (p = 0.035), while weight increased by 4.4 kg at 12 months and remained stable thereafter (p < 0.001 at both time points). The median number of IV antibiotic courses per year decreased from one to none, while ppFEV1 increased by 15 points (p < 0.001) at 12 months and remained stable at 24 months (see Table 2).

**Table 2:** Comparison between baseline and M12 and M24 among ETI-treated and unexposed CFRD adults (n = 49).

	CFRD adults not treated with ETI (n = 10)		CFRD adults treated with ETI (n = 39)	
	Mean difference (95 % CI)	p value	Mean difference (95 % CI)	p value
Insulin dose-adjusted hemoglobin A1c^†^				
M12	−0.79 (−2.09 to 0.52)*	0.230	−0.87 (−1.39 to −0.36)	<0.001
M24	−0.66 (−1.96 to 0.65)*	0.313	−1.44 (−1.96 to −0.91)	<0.001
Hemoglobin A1c (%)				
M12	−0.40 (−1.11 to 0.31)	0.261	−0.55 (−0.87 to −0.26)	<0.001
M24	−0.31 (−1.02 to 0.40)	0.381	−0.65 (−0.96 to −0.34)	<0.001
Hemoglobin A1c (mmol/mol)				
M12	−4.37 (−12.1 to 3.39)	0.261	−5.86 (−9.30 to 2.42)	0.001
M24	−3.39 (−11.2 to 4.38)	0.381	−7.68 (−11.21 to 4.14)	<0.001
Insulin daily dose (U/day)				
M12	−5 (−17 to 6)*	0.344	−1 (−6 to 3)	0.609
M24	−5 (−16 to 7)*	0.418	−5 (−9 to 0)	0.033
Insulin daily dose by weight (U/kg/day)				
M12	−0.10 (−0.27 to 0.08)*	0.978	−0.08 (−0.21 to 0.05)	0.255
M24	−0.26 (−0.26 to 0.09)*	0.324	−0.15 (−0.28 to −0.02)	0.021
Weight (kg)				
M12	1.90 (0.28 to 3.52)	0.026	4.36 (3.01 to 5.71)	<0.001
M24	1.70 (0.08 to 3.32)	0.044	4.44 (3.08 to 5.79)	<0.001
CGM measurement, n(%)				
M12	0	-	12 (31 %)	<0.001
M24	4 (40 %)	-	19 (49 %)	<0.001
Metformin, n(%)				
M12	2 (20 %)	-	0	<0.001
M24	2 (20 %)	-	0	<0.001
ppFEV1 (percentage points)				
M12	−0.5 (−3.1 to 2.1)	0.701	14.7 (11.4 to 18.0)	<0.001
M24	−0.1 (−2.7 to 2.5)	0.939	16.0 (12.7 to 19.3)	<0.001
IV antibiotic courses per year (n)				
M12	0.00 (−0.86 to 0.86)	1.000	−1.18 (−1.56 to −0.80)	<0.001
M24	0.80 (−0.06 to 1.66)	0.072	−1.18 (−1.56 to 0.80)	<0.001

† calculated as HbA1c (%)/4 x insluin dose (units/kg/day).

* rapid insulin was interrupted in 2 subjects, both of which initiated Metformin, long acting insulin was maintained.

Univariate linear mixed effects model analysis of the difference in means (random coefficient = subject; fixed effect = time;

REML estimation; Bonferroni correction; Satterthwaite method for degrees of freedom).

CFRD: Cystic Fibrosis Related Diabetes; ETI: Elexacaftor-tezacaftor-ivacaftor; med: median.

BMI: Body mass index; HbA1c: glycated hemoglobin; U: Units; CGM: Continuous glucose monitoring; ppFEV_1_: percent predicted Forced Expiratory Volume in one second; IV: intravenous.

Insulin was discontinued in 2 subjects, both of which were CFTR modulator naïve prior to ETI initiation. One was a p.Phe508del/p.Phe508del 25-year-old female, who had had CFRD for 6 years at ETI initiation and had a total insulin daily dose of 7 units per day at baseline. She gained 15 kg over 2 years and her HbA1c decreased from 6.8 % to 6.0 % at 24 months. The other was a 36-year-old female with the p.Phe508del/Q493X genotype who was diagnosed with CFRD 8 years prior to ETI initiation and required 17 units of insulin at baseline. She gained 5 kg and her HbA1c remained stable at 5.7 % throughout the study period. Neither was tested for abnormal glucose tolerance during follow-up.

### Outcomes in the non-treated group

Median [IQR] IDAA1c was somewhat lower initially in the unexposed group at 8.54 [7.91–11.37] and did not change throughout the observation period (p = 0.230 at M12 and p = 0.313 at M24). Weight had increased by almost 2 kg on average at 12 months (p = 0.026) and remained stable at 24 months but no difference in HbA1c or total daily insulin dose was observed, while no change was observed in terms of ppFEV1 and the number IV antibiotics per year (see Table 2). None were taking oral antidiabetic medication at baseline. Two were prescribed Metformin between baseline and 12 months; one subject discontinued rapid-acting insulin and switched to Metformin during the first year of follow-up, maintaining once daily long-acting insulin injections throughout, the other initiated Metformin and continued long and short acting insulin injections.

### Continuous glucose monitoring

At baseline, only 10 of 49 subjects (20 %) had a CGM device. At 24 months of follow-up, the number had increased to 23 (47 %), including 4 in the non-treated group. Mean (±SD) percent time within glycemic target was similar in both groups, at 73 % (±11) in ETI-treated subjects and 71 % (±8) in the unexposed group. None of the included subjects had an automated insulin delivery device either at baseline or thereafter.

### Linear mixed effects model analyses

**Fig. 2:**
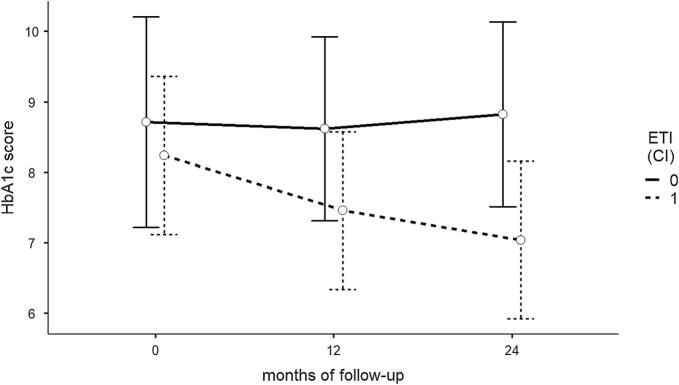
Change in insulin dose adjusted Hemoglobin A1c (IDAA1c) over 12 and 24 months of follow-up in ETI-treated and unexposed CFRD adults (n = 49). Regression slopes from the linear mixed effects model showing the difference in mean IDAA1c between ETI-treated and unexposed subjects at baseline, and after 12 and 24 months of follow-up, including age, sex, time since CFRD diagnosis and Metformin treatment as fixed effects and subject as a random effect. Bars represent mean predictions and 95 % confidence intervals. IDAA1c: insulin dose adjusted hemoglobin A1c; ETI: Elexacaftor-tezacaftor-ivacaftor; CI: 95 % Confidence Interval.

The final LMEM model comparing trends between groups included ‘ETI’, ‘follow-up time’, ‘age at baseline’, ‘sex’, ‘time since CFRD diagnosis’, ‘Metformin’, and an interaction term for ‘ETI:time’ as fixed effects, and ‘subject’ as a random effect. ‘CGM device’ (LRT p = 0.907), and having one or more IV antibiotic courses per year (LRT p = 0.389) did not improve model fit and were not included as parameters in the final model (see Supplementary Table 2). LMEM analysis results showed a numerically relevant association between ETI and decreased IDAA1c, estimated as −1.14 points (95 % CI −2.35 to 0.65) (see Fig. 2). Women and older subjects were more likely to see a decrease in IDAA1c. The introduction of Metformin was correlated with a 3.12 point decrease in IDAA1c (95 % CI −5.02 to −1.21) after controlling for ETI and the other variables but was observed in only two patients, one of which discontinued rapid insulin (see Table 3).

**Table 3:** Linear mixed effects model analysis examining the association between insulin dose adjusted hemoglobin A1c (IDAA1c) and exposure to ETI (n = 49).  Model equation: IDAA1c ∼ 1 + ETI + time + age + sex + time with CFRD  + Metformin + ETI:time + (1|ID).

Parameter estimates (fixed coefficients)	Estimate	Standard Error	95 % Confidence Interval	*t*	*p*
(intercept)	8.14	0.51	7.13 to 9.14	16.05	<0.001
ETI (1–0)	−1.14	0.61	−2.35 to 0.65	−1.87	0.067
M12 vs M0	−0.43	0.30	−1.03 to 0.16	−1.43	0.155
M24 vs M0	−0.54	0.31	−1.16 to 0.08	−1.72	0.088
Age at M0	−0.07	0.03	−0.12 to −0.02	−2.67	0.011
Women vs men	−1.08	0.50	−2.07 to −0.09	−2.17	0.036
Time with CFRD	−0.01	0.04	−0.09 to 0.08	−0.13	0.894
Metformin	−3.12	0.96	−5.02 to −1.21	−3.23	0.002
ETI:M12	−0.70	0.60	−1.87 to 0.48	−1.17	0.245
ETI:M24	−1.32	0.60	−2.50 to −0.14	−2.21	0.030

Results of the final model fitted using a stepwise maximal to minimal approach. Model fit tested using Akaïke lnformation Criterion scores and Likelihood Ratio Tests, checking for model convergence and normal distribution of residuals. Statistical significance tests for model coefficients were performed using Satterthwaite approximations. Restricted Maximum Likelihood estimations. All parameters considered as simple measures.

*Number of observations: 143, Number of groups: 49*.

ETI: Elexacaftor-tezacaftor-ivacaftor; CFRD: Cystic Fibrosis Related Diabetes; IV: intravenous.

The subgroup analysis in the ETI-treated group showed that the decrease in IDAA1c reached −0.77 points (95 % CI: −1.92 to 0.03) at 12 months and −1.29 points at 24 months (95 % CI: −1.83 to −0.75) after adjusting for prior modulator exposure, as well as age and sex (see Table 4). As in the first model, having one or more IV antibiotic courses per year, having a CGM device and time since CFRD diagnosis were not found to improve model fit, and were therefore excluded from the final model (see Supplementary Table 3). None of the subjects in this group were treated with Metformin, which was therefore not included as a parameter.

**Table 4:** Linear Mixed Effects Model examining the association between insulin dose adjusted hemoglobin A1c (IDAA1c) and exposure to non-ETI CFTR modulators prior to ETI initiation among CFRD adults (n = 39).  Model equation: IDAA1c ∼ 1 + prior CFTR modulator + time + age + gender (1 | ID).

Parameter estimates (fixed coefficients)	Estimate	Standard Error	95 % Confidence Interval	t	p
(intercept)	8.66	0.24	8.20 to 9.13	36.74	<0.001
Prior modulator (1–0)	−0.95	0.49	−1.92 to 0.03	−1.93	0.062
M12 vs M0	−0.77	0.26	−1.29 to −0.24	−2.91	0.003
M24 vs M0	−1.29	0.27	−1.83 to −0.75	−4.77	<0.001
Age	−0.06	0.03	−0.11 to −0.00	−1.30	0.044
Women vs men	−0.62	0.48	−1.57 to 0.33	−1.30	0.204
Prior modulator: M12	0.70	0.52	−0.34 to 0.74	1.34	0.184
Prior modulator: M24	0.68	0.53	−0.37 to 1.74	1.28	0.204

Results of the final model fitted using a stepwise maximal to minimal backward fitting approach. Model fit tested using Likelihood Ratio Tests and Akaïke Information Criterion scores, checking for model convergence and normal distribution of residuals. Statistical significance tests for model coefficients were performed using Satterthwaite approximations. All parameters considered as simple measures.

*Number of observations: 113, Number of groups: 39*.

ETI: Elexacaftor-tezacaftor-ivacaftor; CFTR: Cystic Fibrosis Transmembrane Regulator; CFRD: Cystic Fibrosis Related Diabetes; M: Month; mod: modulator; IV: intravenous.

## Discussion

In the present observational study, initiation of a treatment with ETI in adults with insulin-treated CFRD was associated with a decrease in IDAA1c by 1.44 points within the first 24 months, reflecting the combined decrease in HbA1c and in total insulin dose, along with concurrent weight gain. Multivariate LMEM analysis comparing ETI-treated subjects to an unexposed group confirmed the effect of ETI on reducing IDAA1c. The decrease in IDAA1c was greater in women compared to men and in older subjects, and time since CFRD diagnosis did not appear to influence the outcome. In the ETI treated group, IDAA1c was one point lower at baseline among those treated with LI or TI compared to CFTR modulator naïve subjects. The subgroup LMEM analysis showed that the effect of ETI on IDAA1c was greater in subjects who were CFTR naïve at ETI initiation but that ETI also led to a further decrease in IDAA1c in those previously treated with TI or LI. This would suggest an additive or synergistic effect of Elexacaftor on previous modulator combinations though the effects of genotype and phenotype cannot be ruled out.

IDAA1c has been used as a clinical outcome measure to evaluate glucose metabolism in Type 1 diabetes [23,[27], [28], [29]] among adults and children but has, to our knowledge, never been used in CFRD. Our choice was motivated by the assumption that HbA1c and daily insulin dose are influenced by the interaction between decreased insulin resistance, due to the decreased frequency of respiratory infectious exacerbations, and increased body weight after the initiation of ETI. Thus, a stable or decreased HbA1c, along with a stable or decreased total daily insulin dose and an increase in weight or BMI, would suggest improved glycemic control. Importantly, our results show that IDAA1c decrease progressed over time, including in subjects previously treated with TI or LI, suggesting that the effect of ETI on glycemic control should be evaluated over several years.

The impact of ETI on weight gain has been clearly established in clinical trials [11,12,[14], [15], [16], [17],^30^] and observational studies [[18], [19], [20],^31^] and has been shown to occur within the first six months and to remain stable thereafter. Our results show an HbA1c decrease of 0.55 percentage points at 12 months and 0.65 points at 24 months in the ETI treated group, and confirm the findings of previous studies [18,19,31]. In a recent prospective observational study that included 434 adults with advanced lung disease, Burgel et al. found a 0.4 percentage point decrease in HbA1c (p < 0.001) 12 months after ETI initiation in a sub-group of 178 adults with CFRD [31]. Several single-center observational studies have reported a similar magnitude of HbA1c improvement along with weight gain [[18], [19], [20]] and stable [18,20] or reduced [19] total insulin daily dose after ETI initiation.

Amini et al. found an increase in weight of 1.5 (±6.6) kg after 2 years of ETI treatment along with stable HbA1c and basal insulin dose in a retrospective study of 27 adults who had had CFRD for a mean duration of 12 (±7) years [18]. Lurquin et al. reported a BMI increase from 20.9 (±1.9) to 22.1 (±3.7) and a decrease in mean (±SD) insulin total daily dose from 50 (±16) U/day to 44 (±20) U/day (p = 0.017) in 17 adults with CFRD after initiation of ETI or TI, though mean time on ETI was only 1.75 months [19]. They also reported a parallel decrease in HbA1c of 0.4 percentage points [19]. CGM data was used by Scully et al. to examine glycemic control after ETI initiation in 17 CFRD patients, and their results show that mean (±SD) percent time spent within glycemic target range (70–180 mg/dL) increased from 63.6 (±5.7) to 74.5 (± .3) (p = 0.011) after 7 months of ETI [20]. Several studies have examined glycemic control after ETI initiation in patients without established CFRD and report improvements in blood glucose levels [32,33] and/or no change in insulin secretion [[33], [34], [35]].

A decrease in IDAA1c could indicate either improved endogenous insulin production or improved insulin sensitivity. The decrease in the frequency of exacerbations, which would decrease systemic inflammation [9], along with improved respiratory function, which would suggest better tolerance to exercise, would point to the latter. However, the increase in body weight and lower IDAA1c that were observed in the ETI treated group are suggestive of improved pancreatic function. Tests for assessing pancreatic reserve and endogenous insulin production, such as C-peptide measurements and mixed meal tolerance tests, were not conducted during routing follow-up in our center but could prove useful in determining the effect of ETI, and the role of the CFTR protein, on β-cell function and secretory capacity. In a single center case-control study of 17 subjects, with abnormal glucose tolerance but not fasting hyperglycemia, Flatt et al. examined glucose-potentiated insulin and C-peptide responses to glucose-potentiated arginine and insulin secretion during mixed-meal tolerance testing before and after ETI initiation [36]. Their results suggest that ETI may improve β-cell processing of proinsulin and meal responsiveness [36]. Evaluating the change in the incidence of CFRD will require further research in pediatric and young adult CF patients after several years of ETI treatment.

Our study has obvious limitations in terms of data collection and interpretation due to the observational and retrospective design. Important confounding factors, such as bioimpedance data, daily carbohydrate intake and physical activity, which could have modified insulin dose requirements, HbA1c and/or weight, could not be included in the analysis as they were not routinely documented in patient files. The use of IDAA1c as an outcome variable led to the exclusion of subjects with CFRD that did not require insulin and of those with missing daily insulin dose data. Recruitment bias therefore limits the external validity of our findings, as these patients may have shown different trends. In addition, a degree of reporting bias cannot be excluded, as the documented insulin dose was that reported by the patient to the clinician and could have been underestimated or underreported after ETI initiation. Documented total insulin daily dose may therefore have been under- or over-estimated and could have been influenced by other factors, such as patient compliance, reported rapid insulin dose injections, occasional dose corrections, internal clinical guidelines and clinician therapeutic habits.

CGM data is considered a more reliable and precise measure of glycemic control than HbA1c in CFRD [37] but CGM devices were not widely reimbursed for people with CF in France prior to the advent of ETI. Only four subjects had a device at the start of the observation period and 23 had one at the 24-month mark. Using CGM data as the main outcome variable would have considerably decreased our sample size and the observation period. Nonetheless, our sample size was more than twice that of other observational studies examining glucose metabolism in adults with established CFRD and was large enough to compare trends between ETI-exposed and unexposed subjects in LMEM analysis. Our results align with previous research and contribute to the body of evidence suggesting ETI is effective in improving glucose control in adults with established CFRD.

Another limit of this study was that the unexposed group was underpowered. Any difference in baseline characteristics, particularly in terms of genotype, could therefore confound the results, which may have been attributed to differences in treatment when they might have been due to underlying differences in disease severity and/or CFTR function. Genotype could not be included as a parameter due to the high degree of collinearity with the exposure variable, as mutations were the main ETI eligibility criteria. However, RCTs and prospective cohort studies evaluating the effect of ETI on the evolution of CFRD are no longer feasible as ETI eligibility criteria have expanded to include all but the rarest CFTR variants, thus reducing the pool of patients unexposed to ETI [38]. Retrospective observational methods are therefore the only feasible design when examining ETI’s effectiveness on clinical parameters that were not explored in RCTs, and selection bias is all but unavoidable.

Before the advent of CFTR modulators, CFRD management consisted in the early introduction of insulin in order to reduce the effects of a catabolic state on respiratory infections, lung function and weight loss [24]. The phenotypic evolution of the ETI treated CF population may alter recommendations and practice and further research is necessary to determine whether HbA1c testing might be a more reliable marker than it has been in the past, and to validate the use of IDAA1c in CFRD. Indeed, better nutritional status, sometimes reaching overweight or obesity, as well as improved respiratory function and overall ageing, will introduce new priorities in CFRD management [13]. IDAA1c, which captures weight, HbA1c and insulin dose, could prove a useful marker for the evaluation of CFRD progression in a population with shifting and complex clinical features.

## CRediT authorship contribution statement

**Espérie Burnet:** Writing – review & editing, Writing – original draft, Methodology, Investigation, Formal analysis, Data curation, Conceptualization. **Deborah Grunewald:** Writing – original draft, Data curation, Conceptualization. **Etienne Larger:** Writing – review & editing, Validation, Methodology. **Florence Camus-Bablon:** Data curation, Conceptualization. **Catherine Eisenhauer:** Methodology, Formal analysis. **François Mifsud:** Methodology. **Clémence Martin:** Investigation. **Isabelle Honore:** Investigation. **Reem Kanaan:** Investigation. **Nicolas Carlier:** Investigation. **Johanna Fesenbeckh:** Investigation. **Helen Mosnier-Pudar:** Validation, Conceptualization. **Pierre-Régis Burgel:** Writing – review & editing, Supervision, Methodology, Investigation, Conceptualization.

## Declaration of competing interest

The authors declare that they have no known competing financial interests or personal relationships that could have appeared to influence the work reported in this paper.
