# Supplementary material for: Decreased insulin dose-adjusted hemoglobin A1c in adults with cystic fibrosis-related diabetes treated with elexacaftor-tezacaftor-ivacaftor
Source: J Clin Transl Endocrinol. 2025 Jul 2;41:100407. doi: 10.1016/j.jcte.2025.100407 (PMC12273438; doi:10.1016/j.jcte.2025.100407)
Supplement: Supplementary Data 1 [file mmc1.docx]

**Supplementary table 1: Baseline characteristics of ETI treated CFRD adults comparing those with prior CFTR modulator treatment to those without (n=39)**

|  | No prior CFTR modulator  (n=22) | With prior CFTR modulator  (n=17)† | p-value |  |
| --- | --- | --- | --- | --- |
| Age (years) | 32 [28-38] | 35 [29-45] | 0.200 |  |
| Time since CFRD diagnosis (years) | 13 [10-18] | 15 [11-18] | 0.504 |  |
| Female sex | 11 (50%) | 8 (47%) | 0.855 |  |
| ppFEV_1_ | 55 [38-79] | 52 [45-76) | 0.561 |  |
| Weight (kg) | 55 [51-60] | 60 [55-65] | 0.207 |  |
| BMI (kg/m²) | 21.2 [20.1-22.3] | 21.5 [19.8-24.0] | 0.533 |  |
| Total daily insulin dose (U/day) | 39 [32-54] | 30 [20-38] | 0.023 |  |
| Total daily insulin dose (U/kg/day) | 0.71 [0.55-0.86] | 0.48 [0.36-0.56] | 0.019 |  |
| Hemoglobin A1c (%) | 6.8 [6.6-7.5] | 6.6 [6.1-7.6] | 0.251 |  |
| Hemoglobin A1c (mmol/mol) | 50.8 [48.6-58.8] | 48.6 [43.2-59.6] | 0.251 |  |
| Insulin dose-adjusted Hemoglobin A1c | 10.2 [9.0-12.1] | 9.1 [7.7-9.4] | 0.011 |  |
| Continuous Glucose Monitoring device | 6 (27%) | 4 (24%) | 0.791 |  |
| IV antibiotic courses / 12 months | 1 [0-3] | 1 [0-2] | 0.882 |  |
| *CFTR* variants  p.Phe508del/p.Phe508del  p.Phe508del/other  Other/other | 13 (59%)  8 (36%)  1* (5%) | 14 (82%)  3 (18%)  0 | <0.258 |  |

Data presented as median [IQR] or n(%). Comparison between groups used the Mann Whitney U test for independent samples or χ^2^ test as appropriate. CF: Cystic Fibrosis; CFRD: Cystic Fibrosis Related Diabetes; ETI: Elexacaftor/Tezacaftor/Ivacaftor; ppFEV1: percent predicted Forced Expiratory Volume in one second

†14 were treated with lumacaftor-ivacaftor, and 3 with tezacaftor-ivacaftor.

* N1303K/N1303K; this patient received ETI as part of the French Compassionate Program.

**Supplementary table 2: Linear Mixed Effects Model maximal to minimal fitting approach for the effect of ETI on Insulin dose adjusted Haemoglobin A1c (IDAA1c) (n=49)**

| **Baseline model: IDAA1c ~ 1 + ETI + time + sex + age + Metformin + CFRD time + CGM device + ≥1 IV antibiotics course/year + ETI:time + ( 1 \| ID )** | | | | | | |
| --- | --- | --- | --- | --- | --- | --- |
| **Parameters removed sequentially (fixed effects)** | **R^2^ conditional** | **R^2^ marginal** | **ΔR^2^** | **LRT**  **p-value** | **AIC** | **Removed from model** |
| *Baseline* | *0.726* | *0.220* | - | *-* | *555* | *-* |
| ≥1 IV atb course/year | 0.724 | 0.220 | 0.002 | 0.389 | 554 | Yes |
| CGM device | 0.721 | 0.221 | 0.003 | 0.907 | 552 | Yes |
| CFRD time | 0.717 | 0.224 | 0.003 | 0.035 | 555 | No |
| Metformin | 0.675 | 0.187 | 0.042 | <0.001 | 555 | No |
| Final model (AIC 545) : IDAA1c ~ 1 + ETI + time + sex + age + CFRD time + Metformin + ETI:time + ( 1 \| ID ) | | | | | | |

ETI: elexacaftor-tezacaftor-ivacaftor; IDAA1c: insulin dose adjusted hemoglobin A1C; CFRD: Cystic Fibrosis Related Diabetes; CGM: Continuous Glucose Monitoring; IV: intravenous; atb: antibiotic; LRT: Likelihood Ratio Test; AIC: Akaïke lnformation Criterion

*Final model with `Subject ID` as random coefficient, Reduced maximal likelihood estimation of parameters applied. Normality of residuals tested using the Shapiro-Wilk test.

All likelihood ratio tests compared the model including the tested parameter to the model without, in sequence. Parameters removed if AIC after parameter removal < AIC of previous model and if LRT p>0.05.

Interpretation: Sequential removal of ≥1 IV antibiotic courses per year, CGM device and CFRD time were found to improve model fit. Removal of Metformin did not.

**Supplementary table 3: Linear Mixed Effects Model maximal to minimal fitting approach for the effect of prior CFTR modulator on Insulin dose adjusted Haemoglobin A1c after ETI initiation (n=39)**

| **Baseline model: IDAA1c ~ 1 + Prior CFTR modulator + time + age + sex + CFRD time + CGM device + ≥1 IV antibiotics/year + Prior CFTR modulator:time + ( 1 \| ID )** | | | | | | |
| --- | --- | --- | --- | --- | --- | --- |
| **Parameters removed sequentially (fixed effects)** | **R^2^ conditional** | **R^2^ marginal** | **ΔR^2^** | **LRT**  **p-value** | **AIC** | **Removed from model** |
| *Baseline* | *0.678* | *0.303* | - | *-* | *433* | *-* |
| ≥1 IV atb course/year | 0.672 | 0.305 | 0.011 | 0.307 | 432 | Yes |
| CGM device | 0.666 | 0.309 | 0.006 | 0.619 | 430 | Yes |
| CFRD time | 0.676 | 0.256 | -0.009 | 0.545 | 428 | Yes |
| Final model (AIC 428): IDAA1c ~ 1 + Prior CFTR modulator + time + age + sex + Prior CFTR modulator:time + ( 1 \| ID ) | | | | | | |

ETI: elexacaftor-tezacaftor-ivacaftor; IDAA1c: insulin dose adjusted hemoglobin A1C; CFRD: Cystic Fibrosis Related Diabetes; CGM: Continuous Glucose Monitoring; IV: intravenous; LRT: Likelihood Ratio Test; AIC: Akaïke lnformation Criterion

*Final model with `Subject ID` as random coefficient, Reduced maximal likelihood estimation of parameters applied. Normality of residuals tested using the Shapiro-Wilk test. All likelihood ratio tests compared the model including the tested parameter to the model without, in sequence. Parameters removed if AIC of model including parameter > AIC of nested model and if LRT p>0.05.

Interpretation: Sequential removal of ≥1 IV antibiotic courses per year, CGM device and CFRD time were found to improve model fit. Removal of Metformin did not.
